# Community data-driven approach to identify pathogenic founder variants for pan-ethnic carrier screening panels

**DOI:** 10.1186/s40246-023-00472-w

**Published:** 2023-03-28

**Authors:** Yaron Einhorn, Moshe Einhorn, Alina Kurolap, Dror Steinberg, Adi Mory, Lily Bazak, Tamar Paperna, Julia Grinshpun-Cohen, Lina Basel-Salmon, Karin Weiss, Amihood Singer, Yuval Yaron, Hagit Baris Feldman

**Affiliations:** 1 Genoox, Tel Aviv, Israel; 2 The Genetics Institute and Genomics Center, Tel Aviv Sourasky Medical Center, 6 Weizmann St., Tel Aviv, Israel; 3 Beilinson Hospital, Rabin Medical Center, Recanati Genetics Institute, Petah Tikva, Israel; 4 Rambam Health Care Campus, The Genetics Institute, Haifa, Israel; 5 Community Genetic Services, Ministry of Health, Tel Aviv, Israel; 6 Sackler Faculty of Medicine, Tel Aviv University, Tel Aviv, Israel; 7 The Ruth and Bruce Faculty of Medicine, Technion – Israel Institute of Technology, Haifa, Israel

**Keywords:** ACMG, Carrier screening, Community data-driven approach, Genomics, Pan-ethnic, Pathogenic founder variants

## Abstract

**Background:**

The American College of Medical Genetics and Genomics (ACMG) recently published new tier-based carrier screening recommendations. While many pan-ethnic genetic disorders are well established, some genes carry pathogenic founder variants (PFVs) that are unique to specific ethnic groups. We aimed to demonstrate a community data-driven approach to creating a pan-ethnic carrier screening panel that meets the ACMG recommendations.

**Methods:**

Exome sequencing data from 3061 Israeli individuals were analyzed. Machine learning determined ancestries. Frequencies of candidate pathogenic/likely pathogenic (P/LP) variants based on ClinVar and Franklin were calculated for each subpopulation based on the Franklin community platform and compared with existing screening panels. Candidate PFVs were manually curated through community members and the literature.

**Results:**

The samples were automatically assigned to 13 ancestries. The largest number of samples was classified as Ashkenazi Jewish (*n* = 1011), followed by Muslim Arabs (*n* = 613). We detected one tier-2 and seven tier-3 variants that were not included in existing carrier screening panels for Ashkenazi Jewish or Muslim Arab ancestries. Five of these P/LP variants were supported by evidence from the Franklin community. Twenty additional variants were detected that are potentially pathogenic tier-2 or tier-3.

**Conclusions:**

The community data-driven and sharing approaches facilitate generating inclusive and equitable ethnically based carrier screening panels. This approach identified new PFVs missing from currently available panels and highlighted variants that may require reclassification.

**Supplementary Information:**

The online version contains supplementary material available at 10.1186/s40246-023-00472-w.

## Background

Carrier screening aims to identify individuals or couples at risk of having offspring affected with a serious heritable autosomal recessive or X-linked disorder. Couples at risk can then receive personalized counseling regarding their risk and consider their reproductive options, such as prenatal diagnosis or preimplantation genetic testing.

The 2015 recommendations for carrier screening by the American College of Medical Genetics and Genomics (ACMG) focused on a limited number of genes and conditions [1]. In recent years, the rapid development of high-throughput next-generation sequencing (NGS) and its decreasing costs have allowed the simultaneous identification of sequence variants across many genes. As such, NGS has made carrier screening more widely accessible for a wide range of conditions in diverse populations. As a result, the ACMG recently proposed precise tier-based recommendations for carrier screening [2]. Carrier screening based on previous recommendations now falls in tiers 1 & 2, which include a subset of recommended genes for screening by the ACMG and the American College of Obstetricians and Gynecologists, and genes with a carrier frequency of ≥ 1/100, respectively. Two additional screening tiers were added: tier 3 recommends carrier screening for variants with a carrier frequency of ≥ 1/200 and variants for X-linked conditions for all pregnant patients and those planning a pregnancy, while tier 4 recommends screening for variants with a carrier frequency < 1/200 when a pregnancy stems from a known or potential consanguineous relationship, or when warranted by family or personal medical history [2].

Previous screening recommendations were based on self-reported ancestry [1], which is error-prone and often biased. Therefore, the ACMG now recommends that carrier screening should be ethnicity- and population-neutral, and more inclusive of diverse populations [2]. While many pan-ethnic conditions are well-established, some genes carry pathogenic founder variants (PFVs) that are prevalent only in specific ethnic groups; such PFVs may not be assigned to the correct tier or may not be represented at all in the general carrier screening panels. Moreover, while the majority of ethnic groups are usually well-characterized, minority ethnic subpopulations are often less investigated, and thus, PFVs relevant to them may not be considered. Pan-ethnic carrier screening panels with better representation of different ancestries are required to improve carrier screening.

We developed a community data-driven pipeline for creating a pan-ethnic carrier screening panel on top of Franklin data analysis platform (Genoox, Tel Aviv, Israel) [3]. Community data from 150,000 samples from various populations have been uploaded to the Franklin data analysis platform. The platform has been used in over 100 studies at genetics institutes around the world, varying from resolving variants in individual patients [4, 5], to automatically classifying variants from clinical variation databases [6], and identifying rare disease-associated variants in large patient cohorts [7, 8]. Moreover, the platform has been used clinically to analyze NGS data for several years in hundreds of genetics institutes worldwide, including in Israel. The current study aimed to demonstrate the usefulness of a community data-driven approach to create a pan-ethnic carrier screening panel that meets the new ACMG recommendations. The specific study's objectives were to: (1) apply the community data-driven pipeline to the Israeli population, which is ethnically diverse, and determine ancestry-specific carrier frequencies; (2) identify new PFVs that were previously missed and should be added to current screening panels; and (3) demonstrate the crucial role of sharing clinical evidence between community members to remove incorrectly classified variants and confirm true PFVs.

## Materials and methods

### Dataset

The initial dataset included de-identified exome sequencing (ES) data from 6242 Israeli individuals from Tel-Aviv Sourasky Medical Center and Rambam Health Care Campus. All genetic testing was performed as a clinical service under standard clinical consent. The study was approved by the Tel Aviv Sourasky Medical Center institutional review board (no. 0039-15-TLV) and the Rambam Health Care Campus institutional review board (RMC-D-0259-22). Retrospective data collection from patients’ records was granted a waiver of informed consent, as all clinical data contained in this report have been de-identified.

**Fig. 1:**
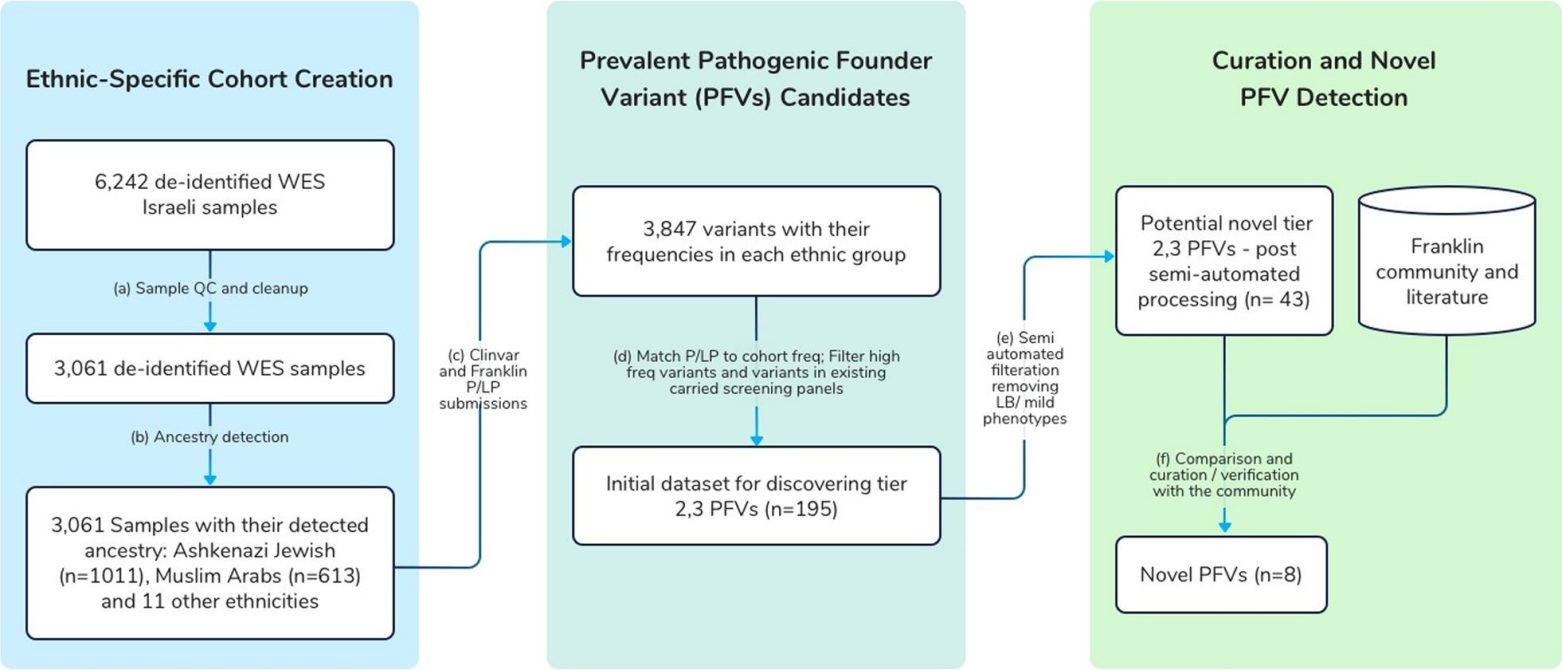
Pipeline for generation and application of a carrier screening panel. Arrows are used to indicate each processing step, and rectangles represent data generated after each step. Left panel: Ethnicity-specific cohort creation—initial dataset of 6242 exomes. **a** Quality control (QC) and related samples removal resulted in 3061 samples; **b** a machine learning model performed ethnicity/ancestry detection, as detailed in the methods section. The two largest ancestry groups were Ashkenazi Jewish (1013 samples) and Muslim Arabs (613 samples), as well as 11 additional inferred ethnicities with smaller numbers of samples. Middle panel: Prevalent PFV candidates—**c** intersection of the cohort variants with ClinVar and Franklin Community submissions resulted in 3847 reported P/LP variants. Variant frequencies were calculated per each ancestry for each of these P/LP variants; **d** in order to focus on novel PFVs only, variants present in existing carrier screening panels were removed. Removal of variants with a carrier frequency less than 1/200 in the Ashkenazi Jewish or Muslim Arab ancestries resulted in 195 candidate tier 2 or 3 variants (Additional file 1: Table S2). Right panel: Curation and novel PFV detection—**e** a semi-automated process to filter out variants with an overall gnomAD frequency > 0.5% or with three or more homozygous counts, or variants associated with mild conditions, resulted in 43 strong candidates for novel PVFs; **f** retrieval of real-world evidence from Franklin community members with homozygous samples and evidence from the literature resulted in eight novel curated PFVs

Sequencing was performed on a NovaSeq 6000 Sequencer (Illumina, San Diego, CA), X100 paired-end. Quality control steps included removing first- and second-degree relatives as well as duplicated samples based on their kinship coefficients. As the majority of samples were trios, affected probands were removed, and healthy parents were used. A set of 3061 unique ES samples remained for further analysis (Fig. 1).

### Sample analysis

Each sample was processed in order to find carrier P/LP variants. The entire bioinformatics pipeline from FASTQ files to final results was performed using the Franklin genetic analysis platform, as previously described [9]. In brief, we aligned FASTQ files against the GRCh37/hg19 reference genome with BWA version 0.7.17 [10]; variant calling for single nucleotide variants and indels was performed using GATK version 4.0.12.0 and FreeBayes version 1.3.1 [11, 12]. Sequence variant annotation and automated classification were performed using Franklin according to ACMG guidelines [13, 14], data from curated sources (e.g., ClinVar), scientific literature, and Franklin community members’ variant classifications.

### Ancestry inference

Machine learning was used to predict each individual's most likely ancestry so that PFVs could be examined for each subpopulation separately. A subset of 567 of the 3061 samples with self-reported ethnicities was used as a training subset. We performed a principal component analysis with 22,952 common exonic variants; to mitigate bias due to false self-reported ethnicities outlier samples that did not cluster with their reported ancestries were removed. We trained a model using the first ten principal components and then used this model to predict the ancestry of all samples. Details on the algorithms can be found in Additional file 1.

### Candidate PFV selection

Variants reported as P/LP by Franklin community members or ClinVar submissions were selected and compared with variants in an existing carrier screening panel dedicated to the Israeli population that is commonly used [15], in order to find novel PFVs (i.e., pathogenic variants not present in the existing panels). Frequencies of the candidate PFVs were calculated for each of the Israeli subpopulations based on the data of the cohort’s 3061 samples. Additional relevant annotations were used through the Franklin platform, such as variant-specific publications, associated conditions and phenotypes, carrier frequencies, and the number of homozygotes in other populations and the Franklin community database. These annotations were used to exclude variants that are unlikely to be PFVs using a semi-automated process in which variants matching the following conditions were filtered out: an overall gnomAD frequency of > 0.5%; variants with three or more homozygous counts; or variants associated with mild conditions.

To confirm pathogenicity, suspected variants were manually curated using evidence and clinical data from samples in the Franklin community database [16]. Using Franklin community variant matching [16], we contacted Franklin community members that had previously observed these variants in a homozygous state, or members that shared evidence about these variants, to determine whether they were variants of unknown significance (VUS) or bona fide P/LP variants. For accurately calculating the carrier frequency and determining the tier of the final candidate variants, we removed for each variant the samples of parents of probands that were homozygous or compound heterozygous for that variant from the frequency calculations.

### Comparison with existing carrier screening panels

Finally, we compared these variants with existing carrier screening panels that are currently used. Two types of comparisons were performed: (1) four commercial NGS-based carrier screening panels: two Ashkenazi Jewish carrier screening panels with 64 (Sema4, Stamford, CT, USA) [17], and 48 genes (Inheritest, Labcorp, Burlington, NC, USA) [18], and two pan-ethnic carrier screening panels with 502 (Sema4 Elements, Sema4) [19], and 302 genes (Invitae, San Francisco, Ca, USA) [20]; and (2) genotype-based panel which was developed specifically for the Israeli population [15].

## Results

### Novel tier 2 and tier 3 PFV detection

The automatically inferred ancestry grouped the samples into 13 different ancestries (Additional file 1: Table S1). This was done using machine learning with the most probable predicted ancestry with the strongest ethnic indicators (see Additional file 1). The largest number of samples was classified as Ashkenazi Jewish (*n* = 1011), followed by Muslim Arabs (*n* = 613). Note that non-Ashkenazi Jews (such as Sephardic Jews), as well as non-Muslim Arabs, were classified in various separate ancestry groups by machine learning. We focused on the Ashkenazi Jewish and Muslim Arab ancestries due to their larger sample size, making their frequencies and results more accurate. The eleven other ancestry groups were too small to analyze for accurate carrier frequency calculations.

The pipeline started with 3847 variants that had been previously classified as P/LP in the 3061 samples. Initial filtration for reported P/LP variants that were not in the Israeli panel [15] and with carrier frequency ≥ 1/200 in either the Ashkenazi Jewish or Muslim Arab ancestry groups resulted in 195 variants (Additional file 1: Table S2). Additional filtration steps for potential variants that should be included in carrier screening resulted in 25 potential novel PFVs in the Ashkenazi Jewish group and 18 potential PFVs in the Muslim Arab group, i.e., in total, 43 potential PFVs were identified in these two groups (Fig. 1, Additional file 1: Table S3). To confirm pathogenicity, manual curation was done for the 43 potential PFVs using evidence from the Franklin community. We were able to retrieve additional evidence and confirmation of pathogenicity for nine (20.9%) of the 43 variants (Table 1). Six of these nine variants were found in homozygous cases in the Franklin community and were indeed causal for their associated phenotypes, thus confirming their pathogenicity. Three variants were determined to be P/LP based on literature evidence only. Of the additional 34 variants, ten were excluded as they appeared in a homozygous state in healthy individuals or without an associated phenotype. Four additional variants were excluded because they were associated with a mild condition, including stationary night blindness (*GPR179*, MIM #614565), mild cystinuria (*SLC7A9*, MIM #220100), postaxial polydactyly (*IQCE*, MIM# 617642) and pseudoxanthoma elasticum (*ABCC6*, MIM #264800). The remaining 20 variants did not have sufficient evidence to be further classified, thus remaining of uncertain significance and, for now, should not be included in carrier screening panels.

**Table 1:** Eight new pathogenic founder variants identified in two Israeli populations

Population variant	Disease (MIM #)	Evidence	Carrier frequency	Tier
*Ashkenazi Jews*				
*WFS1* NM_006005.3:c.1672C > T; p.Arg558Cys	Wolfram syndrome 1 (#606201)	Five individuals homozygous for this variant were observed in the Franklin community. Three of the patients’ ages ranged between 42 and 49. Phenotypes included Maturity Onset Diabetes of the Young (MODY, in all patients), optic atrophy (in three patients), urinary and fecal incontinence, cerebellar atrophy on MRI, and hearing impairment. This variant was previously reported as a causal variant for Wolfram syndrome with milder phenotypes with only 1/8 presenting with optic atrophy [30]. Our results show that optic atrophy might be more frequent than previously thought (3/5) References: [21, 31]	1/68	2
*PCDH15* NM_001384140.1:c.733C > T; p.Arg245*	Usher syndrome, type 1F (#602083)	Appeared in two affected cases in the Franklin community References: [32, 33]	1/113	3
*DDX11* NM_030653.4:c.1763-1G > C; p.?	Warsaw breakage syndrome (#613398)	Appeared in two affected cases in the Franklin community. It was recently added to the recommended Israeli variants panel (independent of our findings) References: [26, 34]	1/113	3
*EYS* NM_001142800.2:c.9286_9295delGTAAATATCG; p.Val3096Leufs*28	Retinitis pigmentosa 25 (#602772)	Appeared in two affected cases in the Franklin community Reference: [35]	1/113	3
*VPS41* NM_014396.4:c.1984C > T; p.Arg662*	Spinocerebellar ataxia, autosomal recessive 29 (#619389)	A nonsense variant where LOF is the disease mechanism, for which no community evidence was found Reference: [36]	1/113	3
*TKT* NM_001064.4:c.769_770insCTACCTCCTTATCTTCTG; p. Trp257delinsSerThrSerLeuSerSerGly	Short stature, developmental delay, and congenital heart defects (#617044). Also known as: Transketolase deficiency	No community evidence was found Reference: [37]	1/145	3
*CLCN1* NM_000083.3:c.1238 T > G; p.Phe413Cys	Congenital myotonia, autosomal recessive (#255700)	No community evidence was found References: [38, 39]	1/145	3
*Muslim Arabs*				
*ACSF3* NM_001243279.3:c.1470G > C; p.Glu490Asp	Combined malonic and methylmalonic aciduria (#614265)	Appeared in two Muslim Arab cases in the Franklin community as a causal variant Reference: [40]	1/154	3

*MIM* Mendelian Inheritance in Man

We performed a manual inspection of samples of parents of probands that were homozygous or compound heterozygous for a PFV, to improve accuracy of carrier frequency calculations. Upon this inspection, four samples of Muslim Arab parents were removed. These were parents of probands homozygous for NM_003682.4(*MADD)*:c.2816 + 1G > A; p.?, which was a tier 2 variant. Recalculation of the carrier frequency resulted in tier 3. In addition, a single Jewish Ashkenazi parent of a proband compound heterozygous for NM_001384140.1(*PCDH15)*:c.733C > T; p.Arg245* was removed, which did not change its tier.

Overall, the analyses resulted in a final list of eight new tier 2 or tier 3 P/LP variants from either Ashkenazi Jewish (seven PFVs) or Muslim Arab (one PFVs) ethnic groups, which were not yet available in existing Israeli carrier screening panels (Table 1).

### Clinically significant variants in the Ashkenazi Jewish and Muslim Arab ethnic groups

The full list of novel PFVs and their evidence of pathogenicity can be found in Table 1. Of these, a single PFV was a tier 2 variant, and the rest were tier 3. The tier 2 variant, *WFS1* (NM_006005.3): c.1672C > T; p.Arg558Cys, was also found in the Ashkenazi Jewish cohort, with a carrier frequency of 1/67. While this variant had previously been detected in a homozygous state in individuals with Wolfram syndrome (MIM #222300, insulin-dependent diabetes mellitus and optic atrophy), it was reportedly associated with a mild phenotype, and only 1 of 8 cases had optic atrophy [21]. Our results, however, demonstrate that this variant was present in five affected individuals in the Franklin community, all diagnosed with maturity onset diabetes of the young (MODY), three of whom had optic atrophy, and one patient also displayed urinary and fecal incontinence, cerebellar atrophy, and hearing impairment. In addition, one of the PFVs detected, *DDX11* (NM_030653.4):c.1763-1G > C, in association with Warsaw breakage syndrome (MIM #613398), has already been included in the national screening panel in 2021 by the Israeli Ministry of Health [22], further validating our results and methods. An additional potential tier 2 variant, *PTPN23* (NM_015466.4): c.3884_3886delAGA; p.Ala1292del, was found in Ashkenazi Jewish samples with a carrier frequency of 1/53. Variants in *PTPN23* are associated with an autosomal recessive neurodevelopmental disorder and structural brain anomalies with or without seizures and spasticity (MIM #618890). Indeed, through the Franklin community, it was established that a girl with severe neurodevelopmental disease was homozygous for this variant and that the case had been published [23]. Recently (April 2022), a submission for this variant was added to ClinVar (SCV002345885.1), classifying it as benign. After contacting the submitting laboratory, they shared that the evidence for this submission was based on the high frequency in the Ashkenazi Jewish subpopulation, which was considered sufficient for benign classification, without observed evidence from healthy homozygous individuals in their database.

### Variants identified in smaller ancestry groups

All eleven other ancestry groups were each represented by small numbers of individuals (25 to 309) (Additional file 1: Table S1). Consequently, a relatively small number of variants were detected in these groups, and they were too small to estimate true carrier frequencies. Nonetheless, we were able to identify several potential PFVs in these ancestry groups. For example, in the Druze ancestry group, we identified 21 potential PFVs (Additional file 1: Table S4). One of these is the *HBB* (NM_000518.5): c.-136C > G variant, which was detected in four out of 301 individuals (carrier frequency of 1/75). This variant was previously reported in a Syrian Druze family with β-thalassemia (MIM #613985) [24]. However, a larger dataset is required to accurately determine the carrier frequency and the tier level of these potential PFVs in the smaller ancestry groups.

### Comparison with existing NGS-based carrier screening panels

We compared the 43 potential PFVs (the eight confirmed novel PFVs, as well as the remaining candidate 34 VUS/mild phenotype variants, which were reported as P/LP) with variants in commercially available carrier screening panels. Although NGS-based panels are expected to capture more pathogenic variants, they may also yield uncertain or falsely reported variants that can burden the interpretation process, often due to a false report in ClinVar or a submission without detailed evidence. We initially compared the 25 potential Ashkenazi Jewish PFVs we identified with two panels dedicated to the Ashkenazi Jewish population—Sema4 and Labcorp—containing 64 and 48 genes each, respectively (Additional file 1: Table S3). None of the 25 variants (including the seven confirmed novel PFVs) observed in Ashkenazi Jews were present, since the genes were not included in these panels. We also compared the 43 candidate PFVs with two larger pan-ethnic panels—Sema4 and Invitae—that include 502 and 302 genes each, respectively (Additional file 1: Table S3). Only 15 of the variants were present in these panels, of which three were confirmed PFVs, five had benign supporting evidence through the Franklin community, and seven were VUS.

### Validation and confirmation of known PFVs

To assess the sensitivity of our pipeline, i.e., our ability to detect all known tier 2 and tier 3 variants, we used a commonly applied Israeli mutation-based carrier screening panel as a reference set [15]. We identified tier 2 or 3 variants from the existing carrier screening panel by calculating their estimated carrier frequency using an independent dataset taken from the gnomAD Ashkenazi Jewish population dataset and selecting those with a carrier frequency > 1/200. This resulted in a selection of 55 variants, 53 (96%) of which were also included in our initial dataset for candidate PFVs in the Ashkenazi Jewish population (Additional file 1: Table S5). Two of the variants did not occur in our Ashkenazi Jewish dataset—one was a deep intronic variant in the *CFTR* gene, which was not covered in our exome-based dataset, and a second variant was not included in our dataset as it had been previously reported as a VUS rather than pathogenic [25]. Of the 53 overlapping variants, 42 were assigned to tiers 2 or 3 in our dataset (carrier frequency > 1/200), while 11 were in tier 4 with carrier frequency between 1/250 to 1/1011 (Additional file 1: Table S5). These differences in tier classification (tiers 2 and 3 compared with tier 4) can be explained by the sample sizes of the datasets (gnomAD and ours) and by heterogeneity within the Ashkenazi Jewish population [26].

In an additional validation, we compared our 25 final Ashkenazi Jewish potential novel PFVs, and calculated their tier assignment using the independent dataset from gnomAD. Twenty-three of the 25 variants were also assigned to tiers 2 or 3 when using gnomAD frequencies, while the remaining two were tier 4 in gnomAD, but close to tier 3, as they appeared with a carrier frequency of 1/215 (*MYO15A* variant) and 1/216 (*FKRP* variant) (Additional file 1: Table S3).

## Discussion

The latest ACMG recommendations state that “All pregnant patients and those planning a pregnancy should be offered tier 3 carrier screening” and that “carrier screening paradigms should be ethnicity- and population-neutral, and more inclusive of diverse populations to promote equity and inclusion” [2]. These recommendations are expected to strengthen the current trend of both extending the number of genes which should be tested and the scope of the examined variants in each gene, from a limited number of known/common variants to full gene sequencing. Providing such robust tests that support these scopes requires an evidence-based and careful curation process to avoid disclosure of VUS or likely benign variants while making sure no proper P/LP variants are misclassified.

Carrier screening is well established and widely used in Israel, and a wealth of knowledge has accumulated throughout the years on ethnically based pathogenic variants, which are major strengths for our study. The Ministry of Health (MOH) established the Israeli genetic carrier screening program as early as the 1980s [27–29]. The program gradually expanded over the years to include tier 1 testing and is offered to all couples in Israel based on their ethnic backgrounds and provided without out-of-pocket expense. Tier 2 tests are subsidized for 80% of the population who acquire supplemental health insurance. Thus, the wide availability of tier 1 & 2 carrier testing made Israel a unique place for the current study. The valuable insights gained from tier 1 & 2 testing serve as the basis for evaluating the impact of the recent ACMG recommendations. Furthermore, the data accumulated via clinical exome testing over the last five years allowed us to compare the current tier 1 & 2 testing platform against real-world NGS data. The key focus of this work was to develop a pipeline for creating pan-ethnic carrier screening panels following the recent ACMG recommendations and to demonstrate its clinical utility and validation by comparison to well-established commercial carrier tests. We show that a data-driven pipeline, which leverages real-world data, is crucial for establishing a complete and accurate panel. With a relatively small dataset, we managed to detect > 98% of the variants being tested today and identify additional variants which may warrant further review and consideration regarding their inclusion in future carrier screens. Moreover, a substantial contribution from the Franklin community supported benign evidence for candidate variants. Contacting community members facilitated the decision of whether a variant should be included or excluded (e.g., due to mild phenotypes, low penetrance, or benign classification). This shows that public-only datasets, and even commercially available tests, may not be accurate enough to determine the best composition of a carrier screening panel.

Specifically, our results show that NGS allows for a standardized platform for the diverse Israeli ethnic subpopulations and can unveil novel tiers 2 and 3 P/LP PFVs. When comparing our findings with existing carrier screening panels for the Ashkenazi Jewish population, we noted that none of the seven novel Ashkenazi Jewish PFVs we observed were included in the existing panels, showing the limitations of targeted carrier screening panels even in well-studied populations. In addition, even when comparing the eight novel PFVs (including both Ashkenazi Jewish and Muslim Arab) against full-gene expanded carrier screening panels, six variants (corresponding to six genes) were not included in these panels. The additional three PFVs, which were covered in the pan-ethnic panels, show the sensitivity advantage when using NGS panels.

Conversely, when comparing the 43 potential PFVs with variants in various commercially available carrier screening panels, we found that these pan-ethnic panels included 12 VUS variants, five of which had benign supporting evidence through the Franklin community but were reported as P/LP by another clinical laboratory or in the literature. These findings demonstrate the additional workload and possibly false-positive reports that can derive from whole-gene NGS-based panels. Similarly, the case of the *PTPN23* potentially novel PFV, which was classified as benign in ClinVar, demonstrates how PFVs can potentially be erroneously classified, solely based on their high frequency in certain subpopulations, which is not uncommon for PFVs. These potential misclassifications can be reduced by evidence-sharing and continuous investigation of candidate PFVs, until reaching a consensus.

This study has several limitations. First, the determined carrier frequencies may be somewhat overestimated as the samples were derived from affected families. However, as the families came for a variety of conditions, this limitation is unlikely to affect the identification of specific PFVs, but may affect their assignment to a specific tier. Second, although we used a large number of unique unrelated samples (*n* = 3061), these were derived from individuals of various ancestries. As such, most ethnic groups were not large enough to accurately estimate carrier frequencies, and consequently, we only estimated carrier frequencies in the two largest groups (Ashkenazi Jews and Muslim Arabs). This limitation will be overcome with time as the numbers of ES tests in the community database are growing, which will eventually allow the analysis of the less common ethnic groups. Also, our study did not account for mixed ancestries and used the most probable ethnicity assignment, which may impact the ancestry-specific frequencies. Therefore, the carrier frequencies might be somewhat higher or lower in these subgroups. Nevertheless, to verify our results and tier assignment, we calculated the carrier frequency of our eight new PFVs in Ashkenazi Jews using the Ashkenazi Jewish subpopulation in the gnomAD database, which resulted in similar tier assignments and validated our results. Third, our approach is based on large numbers of previously identified variants combined with evidence from a community database, meaning that this approach is not specifically geared to the identification of novel P/LP variants in known disease-causing genes or potential P/LP variants in novel genes. However, this limitation also exists for currently employed screening panels and thus is not unique to the approach we developed. Last, this study relied on an ES dataset which precludes the analysis of intragenic or deep noncoding regions and structural variations. This limitation can be overcome with the increasing use of genome sequencing.

In conclusion, we demonstrated the proof-of-concept utility of a community data-driven pipeline that meets the current ACMG recommendations in two common Israeli ethnic groups. We demonstrated that our pipeline can identify various PFVs, including those not yet incorporated in existing Israeli carrier screening panels. Moreover, this approach facilitated the proper reclassification of variants in existing screening panels. As was shown for the Israeli population, the Franklin pipeline is available for developing pan-ethnic carrier screening panels in other countries. With this successful proof-of-concept NGS-based expanded carrier screening, and the foreseeable expansion of the community database, we will be able to apply our panel to all Israeli subpopulations to create an inclusive and equitable carrier screening panel.

## Supplementary Information

**Additional file 1**. **Supplementary Methods.** Descripton of ancestry inference method. **Table S1.** Distribution of ancestries in the Israeli cohort. **Table S2.** 195 P/LP variants with carrier frequency ≥ 1/200 in either the Ashkenazi Jewish or Muslim Arab ancestry groups that are present in existing carrier screening panels. **Table S3.** The 43 potential novel PFVs with carrier frequency ≥ 1/200 in the Ashkenazi Jewish and Muslim Arab ancestry groups. **Table S4.** Potential novel PFVs with carrier frequency ≥ 1/200 in the Druze ancestry group. **Table S5.** The 55 variants in existing carrier screening panels that are in Tier 2 or Tier 3 based on gnomAD frequencies.

## Data Availability

The dataset supporting the conclusions of this article is included within the article and Additional file 1. Identifiable participant information is not publicly available in accordance with research ethics policies.

## Abbreviations

ACMG: American College of Medical Genetics and Genomics
ES: Exome sequencing
MODY: Maturity onset diabetes of the young
MOH: Ministry of Health
NGS: Next-generation sequencing
PFV: Pathogenic founder variants
VUS: Variants of unknown significance
