# Supplementary material for: Community data-driven approach to identify pathogenic founder variants for pan-ethnic carrier screening panels
Source: Hum Genomics. 2023 Mar 28;17:30. doi: 10.1186/s40246-023-00472-w (PMC10044388; doi:10.1186/s40246-023-00472-w)
Supplement: Supplementary file 1 — Additional file 1. Supplementary Methods. Descripton of ancestry inference method. Table S1. Distribution of ancestries in the Israeli cohort. Table S2. 195 P/LP variants with carrier frequency ≥ 1/200 in either the Ashkenazi Jewish or Muslim Arab ancestry groups that are present in existing carrier screening panels. Table S3. The 43 potential novel PFVs with carrier frequency ≥ 1/200 in the Ashkenazi Jewish and Muslim Arab ancestry groups. Table S4. Potential novel PFVs with carrier frequency ≥ 1/200 in the Druze ancestry group. Table S5. The 55 variants in existing carrier screening panels that are in Tier 2 or Tier 3 based on gnomAD frequencies. [file 40246_2023_472_MOESM1_ESM.docx]

# Supplementary materials

## **Supplementary Methods**

### Ancestry inference

Out of 3,061 samples, 567 samples had reported ancestries and were used as the basis for inference for all samples in the cohort. This resulted in recognition of 13 different ancestry types (**Table S1**).

In order to infer ancestry, we selected a subset of common exonic variants using the gnomAD database, similar to previous methods [1]. We annotated gnomAD variants using the Franklin annotation engine and RefSeq transcripts. We filtered only variants located in the coding areas of autosomal chromosomes, which pass the gnomAD QC criteria, with an overall minor allele frequency between 0.04 to 0.96. This resulted in a subset of 22,952 common exonic variants. Next, we extracted the genotypes of the 3,016 samples for the 22,952 variants and performed a principal component analysis using Hail for the 567 samples with their reported ancestry [2]. We used visual inspection of PCA-UMAP plots to remove outlier samples which did not cluster with their reported ancestries [3]. We then selected the first 10 principal components as features, and trained a random forest model with a balance subsampling using the remaining samples as the training dataset. Finally, using our trained model, we predicted the ancestry of all 3,016 samples, using the most probable predicted ancestry for the study.

**Supplementary Tables**

**Table S1. Distribution of ancestries in the Israeli cohort**

| **Ancestry** | **Number of individuals** |
| --- | --- |
| Ashkenazi Jewish | 1,011 |
| Muslim Arab | 613 |
| Iraqi Jewish | 309 |
| Druze | 301 |
| Moroccan Jewish | 290 |
| Bedouin Arab | 125 |
| Russian Jewish | 114 |
| Christian Arab | 103 |
| Caucasian Jewish | 60 |
| Ethiopian Jewish | 54 |
| Iranian Jewish | 30 |
| Bukharan Jewish | 26 |
| Yemeni Jewish | 25 |
| **Total** | **3,061** |

## **Table S2. 195 P/LP variants with carrier frequency ≥1/200 in either the Ashkenazi Jewish or Muslim Arab ancestry groups that are present in existing carrier screening panels**

|  |  |  | **Carrier frequency, 1/x** | |
| --- | --- | --- | --- | --- |
| **Gene name** | **Genetic variation** | **Protein change** | **AJ, x** | **MA, x** |
| *MT-ND5* | ENST00000361567.2:c.370T>C | p.Phe124Leu | 4.9 | 2.7 |
| *PRSS1* | NM_002769.5:c.47C>T | p.Ala16Val | 9.6 | 6 |
| *KRT6B* | NM_005555.4:c.1495G>A | p.Gly499Ser | 10 | 17.5 |
| *CFTR* | NM_000492.4:c.1210-11T>G |  | 10.9 | 19.1 |
| *NOX1* | NM_007052.5:c.721C>T | p.Arg241Cys | 11.2 | 307 |
| *FKBP10* | NM_021939.4:c.917+53G>T |  | 15.4 | 14.8 |
| *AMPD1* | NM_000036.3:c.959A>T | p.Lys320Ile | 19.2 | 36.6 |
| *RBM8A* | NM_005105.5:c.-21G>A |  | 20.3 | 13.8 |
| *FCN3* | NM_003665.4:c.349delC | p.Leu117Serfs*65 | 21.6 | 16.6 |
| *RELT* | NM_152222.2:c.1264C>T | p.Arg422Trp | 22.5 | 613.5 |
| *F5* | NM_000130.5:c.1601A>G | p.Gln534Arg | 23 | 5.8 |
| *PRSS1* | NM_002769.5:c.86A>T | p.Asn29Ile | 24 | 15.8 |
| *TGM4* | NM_003241.4:c.806G>A | p.Trp269* | 24 | 17.5 |
| *GLUD2* | NM_012084.4:c.1492T>G | p.Ser498Ala | 25.2 | 10.3 |
| *CRB2* | NM_173689.7:c.3746G>A | p.Arg1249Gln | 25.2 | 204.8 |
| *REL* | NM_001291746.2:c.992-7C>A |  | 25.8 | 25 |
| *ABCD1* | NM_000033.4:c.1816T>C | p.Ser606Pro | 27.8 | 28.4 |
| *SERPINA1* | NM_000295.5:c.863A>T | p.Glu288Val | 29.4 | 102.7 |
| *VWF* | NM_000552.5:c.5191T>A | p.Ser1731Thr | 30.2 | 88.1 |
| *BCHE* | NM_000055.4:c.293A>G | p.Asp98Gly | 31.1 | 25 |
| *ZGRF1* | NM_018392.5:c.4087G>A | p.Glu1363Lys | 32.1 | 13.6 |
| *ZGRF1* | NM_018392.5:c.142C>A | p.Leu48Met | 32.1 | 18 |
| *SEC63* | NM_007214.5:c.1703_1705delAAG | p.Glu568del | 32.1 | 613.5 |
| *MT-CO1* | ENST00000361624.2:c.1541G>A | p.Ter514Lysext*? | 32.1 | None |
| *PRB3* | NM_006249.5:c.145C>T | p.Arg49Cys | 33.1 | 307 |
| *SCARB1* | NM_005505.5:c.1127C>T | p.Pro376Leu | 33.1 | None |
| *ACKR1* | NM_002036.4:c.265C>T | p.Arg89Cys | 36.6 | 29.7 |
| *SLC7A3* | NM_032803.6:c.1766G>C | p.Ser589Thr | 36.6 | 44.3 |
| *SOD3* | NM_003102.4:c.691C>G | p.Arg231Gly | 39.4 | 23.2 |
| *SLC34A1* | NM_003052.5:c.272_292delTCCCCAAGCTGCGCCAGGCTG | p.Val91_Ala97del | 39.4 | 123.1 |
| *LPL* | NM_000237.3:c.953A>G | p.Asn318Ser | 40.9 | 68.6 |
| *SERPINB8* | NM_002640.4:c.850C>T | p.Arg284* | 42.6 | None |
| *APOC3* | NM_000040.3:c.127G>A | p.Ala43Thr | 42.6 | None |
| *ABCA4* | NM_000350.3:c.4253+43G>A |  | 46.5 | 123.1 |
| *MSR1* | NM_138715.3:c.877C>T | p.Arg293* | 46.5 | 153.8 |
| *MT-ND4* | ENST00000361381.2:c.494T>C | p.Ile165Thr | 46.5 | None |
| *HBD* | NM_000519.4:c.82G>T | p.Ala28Ser | 48.6 | 61.8 |
| *TIMM22* | NM_013337.4:c.97G>C | p.Val33Leu | 48.6 | 77.1 |
| *MT-CYB* | ENST00000361789.2:c.204C>T | p.His68His | 48.6 | 123.1 |
| *ANKRD36* | NM_001354587.1:c.2479-1G>A |  | 51.1 | 88.1 |
| *PRODH* | NM_016335.6:c.1217C>T | p.Pro406Leu | 51.1 | 204.8 |
| *CRB1* | NM_201253.3:c.161G>T | p.Cys54Phe | 51.1 | 613.5 |
| *CEL* | NM_001807.6:c.1776dupC | p.Val593Glyfs*9 | 51.1 | 613.5 |
| *WDR60* | NM_018051.5:c.69G>A | p.Trp23* | 51.1 | None |
| *BDP1* | NM_018429.3:c.7873T>G | p.Ter2625Gluext*? | 53.7 | 123.1 |
| *MPO* | NM_000250.2:c.2031-2A>C |  | 53.7 | 307 |
| *FLG* | NM_002016.2:c.2282_2285delCAGT | p.Ser761Cysfs*36 | 53.7 | 307 |
| *PTPN23* | NM_015466.4:c.3886_3888delAAG | p.Lys1296del | 53.7 | None |
| *MC1R* | NM_002386.4:c.456C>A | p.Tyr152* | 56.7 | 204.8 |
| *PADI3* | NM_016233.2:c.881C>T | p.Ala294Val | 56.7 | None |
| *OCA2* | NM_000275.3:c.1320G>C | p.Leu440Phe | 60 | None |
| *MASP2* | NM_006610.4:c.359A>G | p.Asp120Gly | 63.7 | 77.1 |
| *FLG* | NM_002016.2:c.1501C>T | p.Arg501* | 67.9 | 613.5 |
| *WFS1* | NM_006005.3:c.1672C>T | p.Arg558Cys | 67.9 | None |
| *RP1L1* | NM_178857.6:c.5959C>T | p.Gln1987* | 72.7 | 44.3 |
| *PRODH* | NM_016335.6:c.1322T>C | p.Leu441Pro | 72.7 | 51.6 |
| *KIAA0586* | NM_001329943.3:c.392delG | p.Arg131Lysfs*4 | 72.7 | 307 |
| *PKHD1* | NM_138694.4:c.3766delC | p.Gln1256Argfs*47 | 78.3 | 613.5 |
| *CDHR1* | NM_033100.4:c.783G>A | p.Pro261Pro | 78.3 | None |
| *TNK2* | NM_001382273.1:c.2864G>A | p.Arg955His | 84.8 | 123.1 |
| *LRRK2* | NM_198578.4:c.6055G>A | p.Gly2019Ser | 84.8 | 204.8 |
| *CTH* | NM_001902.6:c.200C>T | p.Thr67Ile | 84.8 | 307 |
| *KRT18* | NM_000224.3:c.383A>T | p.His128Leu | 84.8 | 307 |
| *CYP21A2* | NM_000500.9:c.-126C>G | p.Val282Leu | 84.8 | 613.5 |
| *FH* | NM_000143.4:c.1431_1433dupAAA | p.Lys477dup | 84.8 | 613.5 |
| *HTT* | NM_002111.8:c.87_110dupGCAGCAGCAGCAGCAGCAGCAGCA | p.Gln30_Gln37dup | 92.4 | 88.1 |
| *COL7A1* | NM_000094.4:c.6527dupC | p.Gly2177Trpfs*113 | 101.6 | 38.8 |
| *MT-CYB* | ENST00000361789.2:c.402G>A | p.Pro134Pro | 101.6 | 41.4 |
| *COQ2* | NM_001358921.2:c.170G>C | p.Ser57Thr | 101.6 | 51.6 |
| *DONSON* | NM_017613.4:c.1466A>C | p.Lys489Thr | 101.6 | 102.7 |
| *MT-ND5* | ENST00000361567.2:c.89delA | p.Asn30Thrfs*7 | 101.6 | 307 |
| *BMP15* | NM_005448.2:c.538G>A | p.Ala180Thr | 101.6 | 613.5 |
| *MYO9A* | NM_006901.4:c.6848G>A | p.Arg2283His | 101.6 | 613.5 |
| *SERPINA1* | NM_000295.5:c.1096G>A | p.Glu366Lys | 101.6 | 613.5 |
| *VWA8* | NM_015058.2:c.2003G>A | p.Arg668Gln | 101.6 | 613.5 |
| *M1AP* | NM_001321739.2:c.797G>A | p.Arg266Gln | 101.6 | None |
| *PCDH15* | NM_001384140.1:c.733C>T | p.Arg245* | 101.6 | None |
| *ABCC6* | NM_001171.6:c.742C>T | p.Leu248Phe | 112.8 | 153.8 |
| *C2* | NM_001282459.2:c.841_868delGTGGACAGGGTCAGGAATCAGGAGTCTG | p.Val281Profs*110 | 112.8 | 307 |
| *SLC7A9* | NM_014270.5:c.544G>A | p.Ala182Thr | 112.8 | 307 |
| *VPS41* | NM_014396.4:c.1984C>T | p.Arg662* | 112.8 | 307 |
| *DRD4* | NM_000797.4:c.235_247delGCCGACCTCCTCC | p.Ala79Serfs*21 | 112.8 | 613.5 |
| *ANOS1* | NM_000216.4:c.1187C>T | p.Ser396Leu | 112.8 | 613.5 |
| *DDX11* | NM_030653.4:c.1763-1G>C | p.? | 112.8 | None |
| *TNFRSF13B* | NM_012452.3:c.204dupA | p.Leu69Thrfs*12 | 112.8 | None |
| *IQCE* | NM_152558.5:c.572_579+1delCCAGCCGCG | p.Pro191Argfs*20 | 112.8 | None |
| *GSS* | NM_000178.4:c.4delG | p.Ala2Profs*14 | 112.8 | None |
| *DMP1* | NM_004407.4:c.135+1G>T |  | 112.8 | None |
| *BRCA1* | NM_007294.4:c.68_69delAG | p.Glu23Valfs*17 | 112.8 | None |
| *EYS* | NM_001142800.2:c.9286_9295delGTAAATATCG | p.Val3096Leufs*28 | 112.8 | None |
| *FKRP* | NM_024301.5:c.1073C>T | p.Pro358Leu | 112.8 | None |
| *F12* | NM_000505.4:c.1681-1G>A |  | 112.8 | None |
| *MT-CYB* | ENST00000361789.2:c.38T>C | p.Leu13Ser | 126.9 | 24.1 |
| *B9D1* | NM_001321217.2:c.473-1G>C |  | 126.9 | 41.4 |
| *SLCO1B1* | NM_006446.5:c.1738C>T | p.Arg580* | 126.9 | 51.6 |
| *PADI3* | NM_016233.2:c.335T>A | p.Leu112His | 126.9 | 68.6 |
| *ABCD1* | NM_000033.4:c.1699C>T | p.Gln567* | 126.9 | 102.7 |
| *ACY1* | NM_000666.3:c.1327C>T | p.Arg443Cys | 126.9 | 153.8 |
| *CYP21A2* | NM_001368143.2:c.844G>T |  | 126.9 | 613.5 |
| *IKBKG* | NM_001099857.5:c.169G>A | p.Glu57Lys | 126.9 | None |
| *EP300* | NM_001429.4:c.6574_6585delCAGCAGCAACAG | p.Gln2192_Gln2195del | 126.9 | None |
| *CYP24A1* | NM_000782.5:c.443T>C | p.Leu148Pro | 126.9 | None |
| *COL1A1* | NM_000088.4:c.3196C>T | p.Arg1066Cys | 126.9 | None |
| *DAAM2* | NM_001201427.2:c.1745C>A | p.Pro582His | 144.9 | 38.8 |
| *MT-ND2* | ENST00000361453.3:c.171C>A | p.Ile57Met | 144.9 | 77.1 |
| *IFT122* | NM_052989.2:c.3487T>A | p.Phe1163Ile | 144.9 | 204.8 |
| *PEX6* | NM_000287.4:c.1802G>A | p.Arg601Gln | 144.9 | 204.8 |
| *RHD* | NM_016124.4:c.809T>G | p.Val270Gly | 144.9 | 204.8 |
| *SLC10A1* | NM_003049.4:c.263T>C | p.Ile88Thr | 144.9 | 307 |
| *POC1A* | NM_015426.5:c.253G>C | p.Val85Leu | 144.9 | 613.5 |
| *MMP20* | NM_004771.4:c.389C>T | p.Thr130Ile | 144.9 | 613.5 |
| *SLC3A1* | NM_000341.4:c.1400T>C | p.Met467Thr | 144.9 | None |
| *PMP22* | NM_000304.4:c.353C>T | p.Thr118Met | 144.9 | None |
| *GPR179* | NM_001004334.4:c.984delC | p.Ser329Leufs*4 | 144.9 | None |
| *TKT* | NM_001064.4:c.769_770insCTACCTCCTTATCTTCTG | p.Trp257delinsSerThrSerLeuSerSerGly | 144.9 | None |
| *CLCN1* | NM_000083.3:c.1238T>G | p.Phe413Cys | 144.9 | None |
| *ABCA4* | NM_000350.3:c.5693G>A | p.Arg1898His | 144.9 | None |
| *APOC3* | NM_000040.3:c.55C>T | p.Arg19* | 169 | 36.6 |
| *BCHE* | NM_000055.4:c.1253G>T | p.Gly418Val | 169 | 44.3 |
| *SMN2* | NM_017411.4:c.859G>C | p.Gly287Arg | 169 | 77.1 |
| *PIEZO1* | NM_001142864.4:c.5773C>T | p.Arg1925Trp | 169 | 123.1 |
| *SERPINA6* | NM_001756.4:c.1165G>A | p.Asp389Asn | 169 | 307 |
| *SLC12A1* | NM_000338.3:c.347G>A | p.Arg116His | 169 | 307 |
| *MYO15A* | NM_016239.4:c.10181C>T | p.Ala3394Val | 169 | 613.5 |
| *RBM8A* | NM_005105.5:c.67+32G>C |  | 169 | None |
| *RMRP* | NR_003051.3:n.239C>T |  | 169 | None |
| *ACO2* | NM_001098.3:c.487G>T | p.Val163Leu | 169 | None |
| *KIRREL1* | NM_018240.7:c.1718C>T | p.Ser573Leu | 169 | None |
| *BCHE* | NM_000055.4:c.635C>T | p.Ala212Val | 169 | None |
| *POMT1* | NM_001077365.2:c.2101dupG | p.Asp701Glyfs*8 | 169 | None |
| *CHEK2* | NM_007194.4:c.470T>C | p.Ile157Thr | 169 | None |
| *TNFRSF13B* | NM_012452.3:c.310T>C | p.Cys104Arg | 202.7 | 102.7 |
| *SCN9A* | NM_001365536.1:c.4645T>C | p.Trp1549Arg | 202.7 | 123.1 |
| *SERPINA1* | NM_000295.5:c.839A>T | p.Asp280Val | 202.7 | 153.8 |
| *MT-ND5* | ENST00000361567.2:c.1301A>G | p.Gln434Arg | 202.7 | 153.8 |
| *SH2B3* | NM_005475.3:c.622G>C | p.Glu208Gln | 253.3 | 29.7 |
| *IGSF3* | NM_001007237.3:c.1724G>A | p.Trp575* | 253.3 | 47.7 |
| *WARS2* | NM_015836.4:c.37T>G | p.Trp13Gly | 253.3 | 56.2 |
| *CD36* | NM_001001548.2:c.1079T>G | p.Leu360* | 253.3 | 61.8 |
| *SBDS* | NM_016038.4:c.258+2T>C |  | 253.3 | 88.1 |
| *PROKR2* | NM_144773.4:c.253C>T | p.Arg85Cys | 337.5 | 123.1 |
| *CRB1* | NM_201253.3:c.498_506delAATTGATGG | p.Ile167_Gly169del | 337.5 | 153.8 |
| *MT-CYB* | ENST00000361789.2:c.85G>A | p.Ala29Thr | 506 | 68.6 |
| *DHTKD1* | NM_018706.7:c.2185G>A | p.Gly729Arg | 506 | 88.1 |
| *GPIHBP1* | NM_178172.6:c.523G>C | p.Gly175Arg | 506 | 88.1 |
| *RNASEL* | NM_021133.4:c.793G>T | p.Glu265* | 506 | 102.7 |
| *SCN10A* | NM_006514.4:c.3674T>C | p.Ile1225Thr | 506 | 123.1 |
| *MPEG1* | NM_001039396.2:c.217A>G | p.Thr73Ala | 506 | 123.1 |
| *DCAF6* | NM_001198956.2:c.2240G>A | p.Arg747Gln | 506 | 123.1 |
| *C6* | NM_000065.5:c.2381+2T>C |  | 506 | 123.1 |
| *G6PD* | NM_001360016.2:c.143T>C | p.Ile48Thr | 506 | 123.1 |
| *GRHL3* | NM_198173.3:c.800C>G | p.Ala267Gly | 506 | 153.8 |
| *ASS1* | NM_054012.4:c.-4C>T |  | 1011.5 | 56.2 |
| *G6PD* | NM_001360016.2:c.202G>A | p.Val68Met | 1011.5 | 56.2 |
| *MYO9A* | NM_006901.4:c.5093A>G | p.Asp1698Gly | 1011.5 | 77.1 |
| *GIGYF2* | NM_001103146.3:c.2378C>T | p.Ala793Val | 1011.5 | 77.1 |
| *PNPT1* | NM_033109.5:c.493C>T | p.Pro165Ser | 1011.5 | 88.1 |
| *DUOX2* | NM_001363711.2:c.1825C>T | p.Pro609Ser | 1011.5 | 88.1 |
| *DMGDH* | NM_013391.3:c.972G>A | p.Trp324* | 1011.5 | 102.7 |
| *FCGR1A* | NM_000566.4:c.274C>T | p.Arg92* | 1011.5 | 123.1 |
| *TARDBP* | NM_007375.4:c.800A>G | p.Asn267Ser | 1011.5 | 123.1 |
| *RHD* | NM_016124.4:c.329T>C | p.Leu110Pro | 1011.5 | 123.1 |
| *PREPL* | NM_001171613.2:c.1753+1G>T |  | 1011.5 | 123.1 |
| *RPS6KC1* | NM_012424.6:c.2710G>A | p.Gly904Ser | 1011.5 | 153.8 |
| *KCNK18* | NM_181840.1:c.414_415delCT | p.Phe139Trpfs*25 | 1011.5 | 153.8 |
| *DPRX* | NM_001012728.1:c.466C>T | p.Arg156* | 1011.5 | 153.8 |
| *ACSF3* | NM_001243279.3:c.1470G>C | p.Glu490Asp | 1011.5 | 153.8 |
| *LAMA5* | NM_005560.6:c.3244C>A | p.Pro1082Thr | 1011.5 | 153.8 |
| *MT-RNR1* | ENST00000389680.2:n.180A>G |  | None | 31.2 |
| *CD36* | NM_001001548.2:c.1156C>T | p.Arg386Trp | None | 51.6 |
| *SPTA1* | NM_003126.4:c.2373C>A | p.Asp791Glu | None | 68.6 |
| *LRRK2* | NM_198578.4:c.4321C>T | p.Arg1441Cys | None | 68.6 |
| *RBBP8* | NM_002894.3:c.298C>T | p.Arg100Trp | None | 68.6 |
| *HBA1* | NM_000558.5:c.95+2_95+6delTGAGG |  | None | 77.1 |
| *GJB2* | NM_004004.6:c.487A>G | p.Met163Val | None | 77.1 |
| *TBX22* | NM_001109878.2:c.459-5T>A |  | None | 88.1 |
| *MT-CYB* | ENST00000361789.2:c.924T>C | p.His308His | None | 88.1 |
| *SERPINA7* | NM_000354.6:c.571G>A | p.Asp191Asn | None | 102.7 |
| *UPB1* | NM_016327.3:c.105-2A>G |  | None | 102.7 |
| *ATP7B* | NM_000053.4:c.1285+5G>T |  | None | 102.7 |
| *MAN2B2* | NM_015274.3:c.112G>A | p.Asp38Asn | None | 123.1 |
| *CD55* | NM_000574.5:c.43delC | p.Leu15Serfs*46 | None | 123.1 |
| *PDHA2* | NM_005390.5:c.679A>G | p.Met227Val | None | 123.1 |
| *FLG* | NM_002016.2:c.5717C>A | p.Ser1906* | None | 123.1 |
| *TTC37* | NM_014639.4:c.4507C>T | p.Arg1503Cys | None | 123.1 |
| *UBN1* | NM_001079514.3:c.2356T>A | p.Leu786Met | None | 153.8 |
| *PCK2* | NM_004563.4:c.577C>T | p.Arg193* | None | 153.8 |
| *KCND3* | NM_001378969.1:c.1348C>T | p.Leu450Phe | None | 153.8 |
| *DCHS1* | NM_003737.4:c.2382G>C | p.Gln794His | None | 153.8 |
| *DDX54* | NM_024072.4:c.856G>A | p.Val286Met | None | 153.8 |
| *MT-ND6* | ENST00000361681.2:c.190A>G | p.Met64Val | None | 153.8 |
| *INSL3* | NM_001265587.2:c.305G>A | p.Arg102His | None | 153.8 |
| *BCAS3* | NM_017679.5:c.1684G>A | p.Gly562Arg | None | 153.8 |
| *MADD* | NM_003682.4:c.2816+1G>A | p.? | None | 153.8 |
| *PADI3* | NM_016233.2:c.1744G>A | p.Ala582Thr | None | 153.8 |

AJ, Ashkenazi Jewish; MA, Muslim Arab; 1/x, carrier frequency

## **Table S3. The 43 potential novel PFVs with carrier frequency ≥1/200 in the Ashkenazi Jewish and Muslim Arab ancestry groups**

| **Ancestry group**  **Gene name** | **Nucleotide change** | **Protein change** | **Carrier frequency, 1/x** | **Classification and evidence** | **Carrier frequency in gnomAD^c^, 1/x** | **Present in AJ carrier screening panel** | **Present in pan-ethnic carrier screening panel** |
| --- | --- | --- | --- | --- | --- | --- | --- |
| **Ashkenazi Jewish** | |  |  |  |  |  |  |
| *CRB1* | NM_201253.3:c.161G>T | p.Cys54Phe | 51.1 | B^a^ | 54.0 | No | Yes |
| *WDR60* | NM_018051.5:c.69G>A | p.Trp23* | 51.1 | VUS | 72.3 | No | No |
| *PTPN23* | NM_018051.5:c.3886_3888delAAG | p.Lys1296del | 53.7 | P/LP^a^ | 39.8 | No | No |
| *BDP1* | NM_018429.3:c.7873T>G | p.Ter2625Gluext*? | 53.7 | B^a^ | 48.8 | No | No |
| *WFS1* | NM_006005.3:c.1672C>T | p.Arg558Cys | 67.9 | P/LP^a^ | 37.8 | No | No |
| *KIAA0586* | NM_001329943.3:c.392delG | p.Arg131Lysfs*4 | 72.7 | B^a^ | 60.5 | No | No |
| *PCDH15* | NM_001384140.1:c.733C>T | p.Arg245* | 101.6 | P/LP^a^ | 115.6 | No | Yes |
| *M1AP* | NM_001321739.2:c.797G>A | p.Arg266Gln | 101.6 | VUS | 110.8 | No | No |
| *DDX11* | NM_030653.4:c.1763-1G>C | p.? | 112.8 | P/LP^a^ | 60.8 | No | No |
| *EYS* | NM_001142800.2:c.9286_9295delGTAAATATCG | p.Val3096Leufs*28 | 112.8 | P/LP^a^ | 197.5 | No | Yes |
| *VPS41* | NM_014396.4:c.1984C>T | p.Arg662* | 112.8 | P/LP^b^ | 104.0 | No | No |
| *FKRP* | NM_024301.5:c.1073C>T | p.Pro358Leu | 112.8 | VUS | 215.5 | No | Yes |
| *GSS* | NM_000178.4:c.4delG | p.Ala2Profs*14 | 112.8 | VUS | 108.4 | No | Yes |
| *TNFRSF13B* | NM_012452.3:c.204dupA | p.Leu69Thrfs*12 | 112.8 | VUS | 89.8 | No | No |
| *ABCC6* | NM_001171.6:c.742C>T | p.Leu248Phe | 112.8 | P/LP, M | 136.9 | No | No |
| *IQCE* | NM_152558.5:c.572_579+1delCCAGCCGCG | p.Pro191Argfs*20 | 112.8 | P/LP, M | 103.2 | No | No |
| *SLC7A9* | NM_014270.5:c.544G>A | p.Ala182Thr | 112.8 | P/LP, M | 82.8 | No | No |
| *EP300* | NM_001429.4:c.6574_6585delCAGCAGCAACAG | p.Gln2192_Gln2195del | 126.9 | VUS | 157.5 | No | No |
| *CLCN1* | NM_000083.3:c.1238T>G | p.Phe413Cys | 144.9 | P/LP^b^ | 199.9 | No | No |
| *TKT* | NM_001064.4:c.769_770insCTACCTCCTTATCTTCTG | p.Trp257delinsSerThrSerLeuSerSerGly | 144.9 | P/LP^b^ | 168.0 | No | No |
| *POC1A* | NM_015426.5:c.253G>C | p.Val85Leu | 144.9 | VUS | 129.5 | No | Yes |
| *SLC3A1* | NM_000341.4:c.1400T>C | p.Met467Thr | 144.9 | VUS | 89.8 | No | Yes |
| *GPR179* | NM_001004334.4:c.984delC | p.Ser329Leufs*4 | 144.9 | P/LP, M | 151.8 | No | No |
| *MYO15A* | NM_016239.4:c.10181C>T | p.Ala3394Val | 169.0 | VUS | 214.6 | No | Yes |
| *RMRP* | NR_003051.3:n.239C>T |  | 169.0 | VUS | 110.3 | No | Yes |
|  |  |  |  |  |  |  |  |
| **Muslim Arab** |  |  |  |  |  |  |  |
| *ASS1* | NM_054012.4:c.-4C>T |  | 56.2 | B^a^ | NR | No | Yes |
| *RBBP8* | NM_002894.3:c.298C>T | p.Arg100Trp | 68.6 | B^a^ | NR | No | No |
| *GJB2* | NM_004004.6:c.487A>G | p.Met163Val | 77.1 | B^a^ | NR | Yes | Yes |
| *MYO9A* | NM_006901.4:c.5093A>G | p.Asp1698Gly | 77.1 | B^a^ | NR | No | No |
| *SBDS* | NM_016038.4:c.258+2T>C |  | 88.1 | B^a^ | NR | No | Yes |
| *DHTKD1* | NM_018706.7:c.2185G>A | p.Gly729Arg | 88.1 | VUS | NR | No | No |
| *ATP7B* | NM_000053.4:c.1285+5G>T |  | 102.7 | VUS | NR | Yes | Yes |
| *UPB1* | NM_016327.3:c.105-2A>G |  | 102.7 | VUS | NR | No | No |
| *TTC37* | NM_014639.4:c.4507C>T | p.Arg1503Cys | 123.1 | B^a^ | NR | No | Yes |
| *MAN2B2* | NM_015274.3:c.112G>A | p.Asp38Asn | 123.1 | VUS | NR | No | No |
| *PDHA2* | NM_005390.5:c.679A>G | p.Met227Val | 123.1 | VUS | NR | No | No |
| *PREPL* | NM_001171613.2:c.1753+1G>T |  | 123.1 | VUS | NR | No | No |
| *ACSF3* | NM_001243279.3:c.1470G>C | p.Glu490Asp | 153.8 | P/LP^a^ | NR | No | Yes |
| *MADD* | NM_003682.4:c.2816+1G>A | p.? | 153.8 | P/LP^a^ | NR | No | No |
| *UBN1* | NM_001079514.3:c.2356T>A | p.Leu786Met | 153.8 | B^a^ | NR | No | No |
| *BCAS3* | NM_017679.5:c.1684G>A | p.Gly562Arg | 153.8 | VUS | NR | No | No |
| *DDX54* | NM_024072.4:c.856G>A | p.Val286Met | 153.8 | VUS | NR | No | No |
| *PCK2* | NM_004563.4:c.577C>T | p.Arg193* | 153.8 | VUS | NR | No | No |

B, benign; M, mild phenotype; NR, not relevant; P/LP, pathogenic/likely pathogenic; VUS, variant of unknown significance; 1/x, carrier frequency.

^a^evidence based on Franklin community. ^b^ evidence based on literature. ^c^ carrier frequency in Ashkenazi Jewish population in gnomAD

## **Table S4. Potential novel PFVs with carrier frequency ≥1/200 in the Druze ancestry group**

| **Gene name** | **Genetic variation** | **Protein change** | **Carrier frequency, 1/x** |
| --- | --- | --- | --- |
| *UBN1* | NM_001079514.3:c.2356T>A | p.Leu786Met | 60.7 |
| *CBS* | NM_000071.3:c.833T>C | p.Ile278Thr | 60.7 |
| *OFD1* | NM_003611.3:c.2927A>C | p.Lys976Thr | 60.7 |
| *MYO9A* | NM_006901.4:c.5093A>G | p.Asp1698Gly | 75.8 |
| *DHTKD1* | NM_018706.7:c.2185G>A | p.Gly729Arg | 75.8 |
| *SERPINA1* | NM_000295.5:c.1177C>T | p.Pro393Ser | 75.8 |
| *CIT* | NM_001206999.2c.1070A>G | p.His357Arg | 75.8 |
| *DUOX2* | NM_001363711.2:c.1126C>T | p.Arg376Trp | 75.8 |
| *ANO5* | NM_213599.3:c.1213C>T | p.Gln405* | 75.8 |
| *MTHFR* | NM_005957.5:c.416C>T | p.Thr139Met | 75.8 |
| *ACY1* | NM_000666.3:c.699A>C | p.Glu233Asp | 75.8 |
| *HBB* | NM_000518.5:c.-136C>G |  | 75.8 |
| *ABCD1* | NM_000033.4:c.1699C>T | p.Gln567* | 100.8 |
| *PCK2* | NM_004563.4:c.577C>T | p.Arg193* | 100.8 |
| *KIAA0586* | NM_001329943.3:c.392delG | p.Arg131Lysfs*4 | 100.8 |
| *PRSS56* | NM_001195129.2:c.1400G>T | p.Arg467Leu | 100.8 |
| *CD36* | NM_001001548.2:c.1144C>T | p.Gln382* | 100.8 |
| *EYS* | NM_001142800.2:c.8168delA | p.Gln2723Argfs*18 | 100.8 |
| *PAH* | NM_000277.3:c.688G>A | p.Val230Ile | 100.8 |
| *CASR* | NM_000388.4:c.848T>C | p.Ile283Thr | 100.8 |
| *TMPRSS3* | NM_001256317.3:c.325C>T | p.Arg109Trp | 100.8 |

1/x, carrier frequency

## **Table S5. The 55 variants in existing carrier screening panels that are in Tier 2 or Tier 3 based on gnomAD frequencies**

|  |  |  | **Carrier frequency in AJ** | |  |
| --- | --- | --- | --- | --- | --- |
| **Gene name** | **Genetic variation** | **Protein change** | **gnomAD, 1/x** | **our data,**  **1/x** | **Tier difference^c^** |
| *CFTR* | NM_000492.4:c.3718-2477C>T |  | 73 | N/A^a^ | N/A |
| *TYR* | NM_000372.5:c.454C>T | p.Pro152Ser | 70 | N/A^b^ | N/A |
| *BCKDHB* | NM_183050.4:c.548G>C | p.Arg183Pro | 94 | 1011 | Yes |
| *MLC1* | NM_015166.4:c.274C>T | p.Pro92Ser | 200 | 1011 | Yes |
| *PHYH* | NM_006214.4:c.823C>T | p.Arg275Trp | 140 | 506 | Yes |
| *FANCC* | NM_000136.3:c.456+4A>T |  | 81 | 337 | Yes |
| *ADAMTS2* | NM_014244.5:c.673C>T | p.Gln225* | 167 | 337 | Yes |
| *BBS2* | NM_031885.5:c.1895G>C | p.Arg632Pro | 179 | 337 | Yes |
| *CRB2* | NM_173689.7:c.2400C>G | p.Asn800Lys | 93 | 253 | Yes |
| *RTEL1* | NM_001283009.2:c.3791G>A | p.Arg1264His | 142 | 253 | Yes |
| *DNAI2* | NM_023036.6:c.1304G>A | p.Trp435* | 148 | 253 | Yes |
| *ATP7B* | NM_000053.4:c.3191A>C | p.Glu1064Ala | 153 | 253 | Yes |
| *LOXHD1* | NM_001384474.1:c.4714C>T | p.Arg1572* | 177 | 253 | Yes |
| *DHDDS* | NM_205861.3:c.124A>G | p.Lys42Glu | 96 | 202 | No |
| *UPB1* | NM_016327.3:c.917-1G>A |  | 136 | 202 | No |
| *TECPR2* | NM_014844.5:c.1319delT | p.Leu440fs | 160 | 202 | No |
| *SMPD1* | NM_000543.5:c.1493G>T | p.Arg498Leu | 178 | 202 | No |
| *MTTP* | NM_001386140.1:c.2674G>T | p.Gly892* | 187 | 202 | No |
| *GMPPB* | NM_021971.4:c.860G>A | p.Arg287Gln | 118 | 169 | No |
| *MCOLN1* | NM_020533.3:c.406-2A>G |  | 120 | 169 | No |
| *OCA2* | NM_000275.3:c.1327G>A | p.Val443Ile | 136 | 169 | No |
| *FAH* | NM_000137.4:c.782C>T | p.Pro261Leu | 136 | 169 | No |
| *COQ4* | NM_016035.5:c.718C>T | p.Arg240Cys | 146 | 169 | No |
| *HIKESHI* | NM_016401.4:c.160G>C | p.Val54Leu | 185 | 169 | No |
| *SLC26A4* | NM_000441.2:c.349C>T | p.Leu117Phe | 96 | 144 | No |
| *DGAT1* | NM_012079.6:c.751+2T>C |  | 103 | 144 | No |
| *THG1L* | NM_017872.5:c.164T>C | p.Val55Ala | 112 | 144 | No |
| *CCDC65* | NM_033124.5:c.877_878delAT | p.Ile293fs | 115 | 144 | No |
| *SMARCAL1* | NM_014140.4:c.863-2A>G |  | 191 | 144 | No |
| *CFTR* | NM_000492.4:c.3454G>C | p.Asp1152His | 192 | 144 | No |
| *DLD* | NM_000108.5:c.685G>T | p.Gly229Cys | 76 | 126 | No |
| *CFTR* | NM_000492.4:c.1521_1523delCTT | p.Phe508del | 89 | 112 | No |
| *ATP7B* | NM_000053.4:c.3207C>A | p.His1069Gln | 94 | 112 | No |
| *PCDH15* | NM_001384140.1:c.733C>T | p.Arg245* | 115 | 101 | No |
| *GJB2* | NM_004004.6:c.35delG | p.Gly12fs | 148 | 101 | No |
| *CFTR* | NM_000492.4:c.3846G>A | p.Trp1282* | 52 | 92 | No |
| *TMEM216* | NM_001173990.3:c.218G>T | p.Arg73Leu | 148 | 92 | No |
| *MMACHC* | NM_015506.3:c.271dupA | p.Arg91fs | 179 | 92 | No |
| *GJB2* | NM_004004.6:c.109G>A | p.Val37Ile | 62 | 72 | No |
| *SLC1A4* | NM_003038.5:c.766G>A | p.Glu256Lys | 106 | 72 | No |
| *FKTN* | NM_001079802.2:c.1167dupA | p.Phe390fs | 64 | 67 | No |
| *CLRN1* | NM_174878.3:c.144T>G | p.Asn48Lys | 87 | 67 | No |
| *MPL* | NM_005373.3:c.79+2T>A |  | 61 | 63 | No |
| *ASPA* | NM_000049.4:c.854A>C | p.Glu285Ala | 54 | 59 | No |
| *G6PC* | NM_000151.4:c.247C>T | p.Arg83Cys | 76 | 59 | No |
| *ABCC8* | NM_000352.6:c.3989-9G>A |  | 84 | 59 | No |
| *TYR* | NM_000372.5:c.1217C>T | p.Pro406Leu | 59 | 56 | No |
| *NCF1* | NM_000265.6:c.579G>A | p.Trp193* | 44 | 51 | No |
| *USH2A* | NM_206933.4:c.12575G>A | p.Arg4192His | 56 | 51 | No |
| *DHCR7* | NM_001360.3:c.964-1G>C |  | 42 | 44 | No |
| *TYR* | NM_000372.5:c.1037-7T>A |  | 33 | 42 | No |
| *ELP1* | NM_003640.5:c.2204+6T>C |  | 37 | 39 | No |
| *HEXA* | NM_000520.6:c.1274_1277dupTATC | p.Tyr427fs | 39 | 39 | No |
| *GJB2* | NM_004004.6:c.167delT | p.Leu56fs | 31 | 36 | No |
| *GBA* | NM_000157.4:c.1226A>G | p.Asn409Ser | 19 | 18 | No |

AJ, Ashkenazi Jewish; N/A, not applicable

^a^ deep intronic variant in the *CFTR* gene, while *CFTR* was not well covered in our dataset. ^b^ not included in our dataset as it had not been reported as pathogenic, only as VUS, and had literature evidence supporting its uncertainty.^7^

^c^Tiers 2 and 3 vs. Tier 4

## **Supplementary References**

1. Karczewski KJ, Francioli LC, Tiao G, Cummings BB, Alfoldi J, Wang Q, et al. The mutational constraint spectrum quantified from variation in 141,456 humans. Nature 2020;581 (7809):434-43. <http://doi.org/10.1038/s41586-020-2308-7>.

2. Hail 0.2. Available from: <https://github.com/hail-is/hail>.

3. Sakaue S, Hirata J, Kanai M, Suzuki K, Akiyama M, Lai Too C, et al. Dimensionality reduction reveals fine-scale structure in the Japanese population with consequences for polygenic risk prediction. Nat Commun 2020;11 (1):1569. 10.1038/s41467-020-15194-z.
